# 

**Published:** 1891-04

**Authors:** 


					﻿Vol. XI.
APRIL, 1891.
NO. 4.
THE
pOMIOPATHlC pHYjSlClAfl,
A Monthly Journal of Homoeopathic Materia Medica
and Clinical Medicine.
” He is a freeman whom truth makes free, and all are slaves beside.”
“ Seek the Truth: come whence it may, cost what it will”
EDITED AND PUBLISHED BY
WALTER M. JAMES, M. ID.,
AND
George h. Clark, m. id.
PHILADELPHIA :
No. 1125 SPRUCE STREKT.
i/oisraDO ar
ALFRED HEATH & CO., Sole Agents for Great Britain,
114 Ebury Street, Eaton Square.
early Subscription, $2.50, invariably in advance. Single numbers, 25 cts.
Entered at the Philadelphia Poet-Office as Second-Class Mail Matter.
				

